# Ultrastructural changes during nectar secretion from extrafloral nectaries of *Pithecellobium dulce* Benth

**DOI:** 10.1007/s00709-023-01853-7

**Published:** 2023-03-23

**Authors:** Pramod Sivan, Karumanchi S Rao

**Affiliations:** 1 Division of Glycoscience, Department of Chemistry, KTH Royal Institute of Technology, AlbaNova University Centre, 106 91 Stockholm, Sweden; 2 Department of Biosciences, Sardar Patel University, Vallabh Vidyanagar, Gujarat 388120 India

**Keywords:** Extrafloral nectaries, Ultrastructure, Developmental changes, Nectar secretion

## Abstract

The structural changes in the secretory cells are important to understand the ontogeny and nectar secretion process from the nectaries. In this study, we investigated the ultrastructural changes during different developmental/secretion stages of extrafloral nectaries (EFNs) of *Pithecellobium dulce.* The dense cytoplasm with active biosynthesis mechanisms such as ribosomes, mitochondria, large nucleus, and plastids with accumulated starch grains characterized the pre-secretion stage of young nectariferous cells. During the secretory phase, the cytoplasm showed distinct changes associated with endomembrane transport such as the predominant occurrence of Golgi, secretory vesicles, and ER resulting in the subsequent appearance of secretions in the intercellular and subcuticular spaces. Cell wall loosening following the dissolution of middle lamellae leading to the formation of subcuticular spaces was evident during advanced stages of nectar secretion. The characteristic cytoplasmic and apoplastic changes associated with cell death were noticed during the post-secretory stages. The structural evidence from the present study suggests the occurrence of two modes of secretion (merocrine and holocrine) during the early and late stages of secretion in the EFNs of *P. dulce.*

## Introduction

The specialized structures found on the plant surface for the secretion of sugar, water, and amino acids are called nectaries (Nicolson and Thornburg 2007; Coutinho et al. 2012). According to the topography, the nectaries associated with floral parts are referred to as floral nectaries, while the glands associated with vegetative portions of plants and which secretes primarily sugars are called extrafloral nectaries (EFNs) (Durkee 1982; Coutinho et al. 2012). EFNs proved to have importance in taxonomic studies and the establishment of phylogenetic relationships within taxonomic groups (Marazzi et al. 2019, Dalvi et al. 2013, 2020; Souza et al. 2021; Coutinho et al., 2022). Furthermore, the chemical nature of the secretion of EFNs has been considered to be an ecologically significant indicator for understanding the relationship between visiting ants and plants (Coutinho et al. 2022). The functional significance of the ontogeny of EFNs is attributed to the support of the mutualists of plants such as ants in *Acacia drepanolobium* (Young et al. 1997) and *Opuntia imbricata* (Miller 2007). The ontogenetic pattern of EFNs has been mostly explained in terms of resource limitation for young plants to produce such traits (Izzo and Vasconcelos 2005; Miller 2007) or due to the architectural constraints associated with the ability of a plant to attract a whole colony of ants (Boege et al. 2007). The occurrence EFNs as complex structures is a characteristic feature of Mimosaceae and is believed to function in attracting aggressive insects such as ants which disturb the herbivores and reduce the damage to plants (Dave and Menon 1987). Previous studies have proposed two methods of nectar secretion based on the cytology of secretory cells; the transport of individual sugar molecules across the secretory cell membrane is called eccrine secretion, whereas the second mode is termed as granulocrine secretion which involves the transport of sugar through vesicles, presumably derived from ER or dictyosomes to the plasmalemma where the fusion of these elements occurs followed by the release of sugar exterior of the nectaries (Fahn and Banouaiche 1979); Durkee 1982).

Due to the attractive role in reproduction, floral nectaries have been studied extensively, while developmental studies of EFNs have received relatively little attention (Delgado et al. 2011; Villamil et al. 2013). The complex biology of EFNs development and their functional importance in various plants have been studied through analysis of various aspects such as emergence, differentiation, senescence, changes in cytoplasm and nuclei density, chemical nature of nectar, the axis of mitotic divisions, and physiological and anatomical constraints limiting EFNs development (Durkee 1982; Thadeo et al. 2008; Villamil et al. 2013). The EFNs mediating ant-plant protection mutualisms are reported to be quite common and unusually diverse in the Leguminosae (Marazzi et al. 2019). Morphological and anatomical characteristics are key indicators of the taxonomic importance of EFNs during plant evolution. For instance, the flat glandular type of EFNs with sap connectivity closer to the phloem of nearby vascular strand are considered to be primitive, while the development of phloem traces into the more definitive glandular structure results in the increase of distance between secretory tissues and vascular strands (Elias 1972). In a recent review on phylogenetic reconstruction of EFNs in leguminosae suggests occurence of diverse EFN types within different genera might have independent evolutionary trajectories (Marazzi et al. 2019). Among different Leguminosae members, Caesalpinioideae group representing maximum morphological diversification is almost exclusively with elevated EFNs, Detarioideae members with flat EFNs, Papilionoideae with swollen scar, and intrastipular EFNs is predominant in Cercidoideae (Marazzi et al. 2019). The genera *Pithecellobium* belongs to Caesalpinioideae. However, the complexity of the nectar secretion process is further increased by the different types of cellular processes, i.e., holocrine (the entire cell disintegrates to release its substances ) and merocrine (glands remain alive and secrete their substances by exocytosis). The majority of recent studies on the EFNs in plants are mainly dealt with their morphology, anatomy, and ecological significance; however, the lacunae of ultrastructural studies hinder our knowledge on the complex biology of the cellular mechanism of nectar secretion in EFNs. This aspect is very critical in unraveling the science behind nectar secretion from EFNs of various species. The genus *Pithecellobium* (Caesalpinioideae) is a large assemblage of tropical and subtropical trees and shrubs. The occurrence of EFNs has been reported in a few species of *Pithecellobium*. *Pithecellobium dulce* is an evergreen medium-sized, spiny tree having vast nutritional and pharmacognosy value for its various parts such as leaves, bark, fruits, and seeds (Kulkarni and Jamakhandi 2018; Murugesan et al. 2019). Although the morphological and anatomical studies in *Pithecellobium* demonstrated the presence of advanced type of EFNs in *P. macradenium* (Elias 1972) and in *P. dulce* (Dave and Menon 1987), but how these phylogenetic advancements are reflected in the EFNs developments, especially the type of glandular secretion in *Pithecellobium dulce*, remain unknown. This information is important to understand the developmental biology of taxonomically advanced type of EFNs in *Pithecellobium* which might have a contributory role in its efficiency in ant-plant mutualism process. Considering the necessity of deciphering the mechanism of nectar secretion, the present study was undertaken to elucidate the ultrastructural changes which occur prior to, during, and post-secretion stages of nectariferous cells in the EFNs of *Pithecellobium dulce*.

## Materials and methods

### Plant material

EFNs of various ages were collected from the rachis of *P. dulce* Benth, growing in the Botanical garden of Sardar Patel University, Gujarat, India. The young nectaries were located on leaves close to the shoot tips, and the mature ones with a tiny droplet of nectar indicating an active secretion stage were located on leaves closer to young leaves. The old and partially dried nectaries without nectar droplets were found on mature leaves away from the shoot tip.

### Sample preparation for microscopy

Samples were fixed in 2.5% glutaraldehyde in phosphate buffer (0.1 M, pH 7.2) for 2 days. They were then post-fixed in 1% osmium tetroxide for overnight, dehydrated in graded acetone series, and embedded in Spurr’s epoxy resin (Spurr 1969).

### Light microscopy

One-micrometer-thick longitudinal sections of the nectaries taken from resin embedded samples were stained with calcofluor white ST (0.1% in distilled water, American Cyanamid Company, Wayne, NY) and observed under a Zeiss fluorescence microscope.

### Transmission electron microscopy

Ultrathin sections were cut on an ultramicrotome (Reichert OM U3, Austria), stained with a saturated solution of uranyl acetate in 50% ethanol for 30 min and in Reynold’s lead citrate for 5 min. Stained sections were examined with a Philips 300 transmission electron microscope.

### Scanning electron microscopy

Samples were dehydrated in ethanol-isoamyl alcohol series, critical-point dried using liquid CO_2_, coated with gold using a sputter coating unit, and observed in a Philips SEM 505 at 10 kv.

## Results

### Structure of nectary

**Fig. 1:**
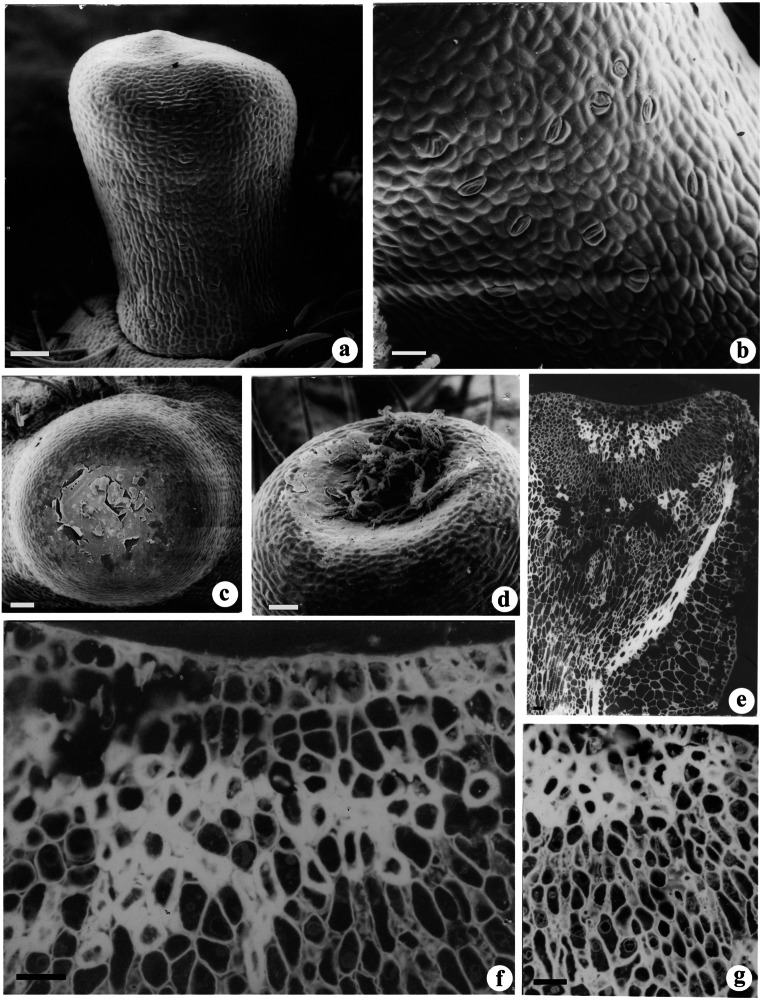
Scanning electron microscopy (**a**–**d**) and fluorescence microscopy (**e**–**g**) images showing surface morphology and internal anatomy of EFNs. **a** The urn shaped nectary at pre-secretory stage. **b** The lateral surface of stalk region showing presence of stomata. **c** The concave surface of mature nectary showing dried nectar oozed out of ruptured cuticle of epidermal cells. **d** The mature nectary after secretion showing the presence of fungal hyphae on the surface. **e** Longitudinally cut nectary showing vascular strand extending from the base to the nectariferous tissue. **f** Secretory tissue in old nectary showing the presence of parenchymatous and sclerenchymatous zones. **g** Enlarged view of F showing sclerenchymatous nectariferous zones. Scale bar: **a**, **c**–**g** = 50 μm, **b** = 20 μm

The small copular (urn-shaped) nectaries in *P. dulce* are generally sessile to sub-sessile with conspicuous rims and cup cavities (Fig. 1a–d). Stomata with guard cells were observed on the lateral surface of the nectary wall (Fig 1b). The dried remains of drops of sticky nectar were noticed in the active nectary cavity (Fig. 1c). The ruptured cuticle was evident on the cup region during secretion stage (Fig. 1c). The mature nectary at post-secretion stage showed the presence of fungal mycelia on the cuticle surface (Fig. 1d). Nectaries are located on the primary rachis between the insertions of each pair of the leaflet. A thick cuticle layer was found on the tangential wall of epidermal cells. Beneath the several layered nectariferous cells zone, vascular traces originated from the petiolar vascular bundles girdle was observed (Fig. 1e). The phloem traces reaches the nectariferous tissue at the tip of the nectary. During the late stages of secretion, the nectariferous cells showed thick wall with intense fluorescence after calcofluor staining indicating apoplastic secretion (Fig. 1f, g). The glandular region of the nectary consists of highly vacuolated cells (Fig. 1g).

### Ultrastructure of nectary

The ultrastructural changes during different developmental stages (pre-secretory, secretory, late secretory, and post-secretory stages) of nectariferous cells in *P. dulce* were observed using transmission electron microscopy.

### Pre-secretory stage

**Fig. 2:**
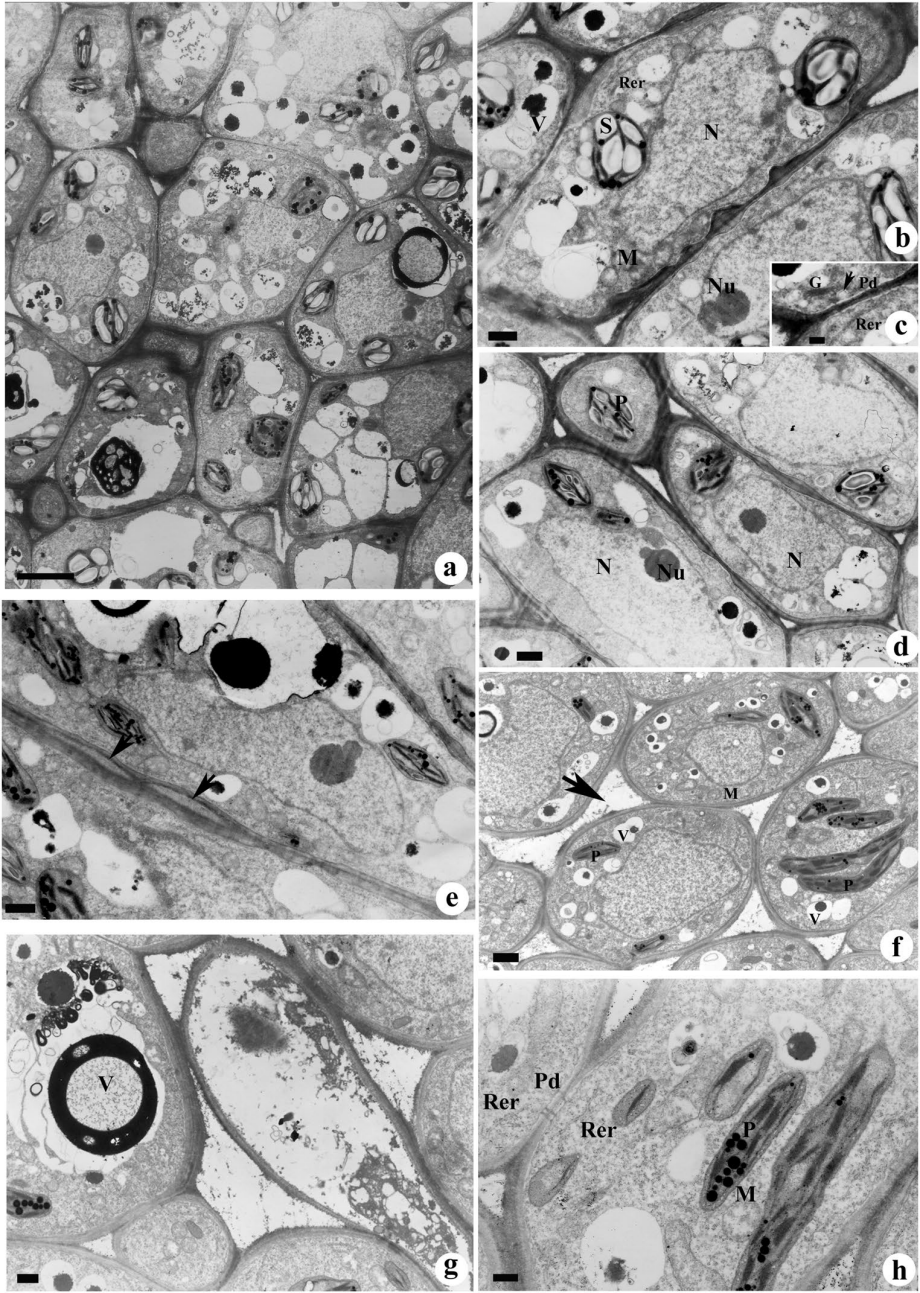
**a**–**h** TEM images of extrafloral nectariferous cells showing ultrastructural changes during the pre-secretory stage. **a** The epidermal and subepidermal nectariferous cells showing dense cytoplasm. **b** The nectariferous cells showing dense cytoplasm containing a large nucleus (N), mitochondria (M), plastid with starch grains (S), and large vacuoles (V) with electron-dense osmophilic bodies. **c** An enlarged view of “b” showing the presence of Golgi bodies (G) and rough endoplasmic reticulum (RER) near the cell wall plasmodesmata region. Arrow indicates the vesicles fused with the plasma membrane. **d** The cell corners showing loosening of middle lamellae prior to nectar secretion. Note the nucleus (N) with nucleolus (Nu) and plastids (P) with plastoglobuli. **e** The cell wall between two adjacent nectariferous cells showing loosening of middle lamellae (arrows). **f** Formation of large intercellular space (arrow) through loosening of middle lamellae. M mitochondria, P plastid, V vacuole. **g** Wide intercellular gap areas formed through the merging of middle lamellae loosened spaces. **h** Fusiform to oval-shaped plastids (P) showing thylakoids and plastoglobuli. Note the presence of rough endoplasmic reticulum (RER) near the plasmodesmata (Pd) and round to oval-shaped mitochondria (M). Scale bar: a =10 μm, b–g = 2 μm

The epidermal and subepidermal nectariferous cells were characterized by dense cytoplasm containing large nuclei, sometimes with more than one nucleolus (Fig. 2a–c) and the presence of long narrow profiles of rough ER cisternae in the periphery of cytoplasm (Fig. 2b, c). Dictyosome in small groups and numerous mitochondria were noticed in these cells (Fig. 2b). Plastids showed the presence of starch grains and plastoglobuli (Fig. 2b). Vacuoles containing electron-dense osmophilic droplets were apparent in the cytoplasm of young nectariferous cells (Fig. 1b, c). The cell walls are irregularly thickened (Fig. 2b, d). The cytoplasmic connection is maintained in the non-thickened areas of the cell wall by plasmodesmata, ER, and RER (Fig. 2c).

### Secretory stage

**Fig. 3:**
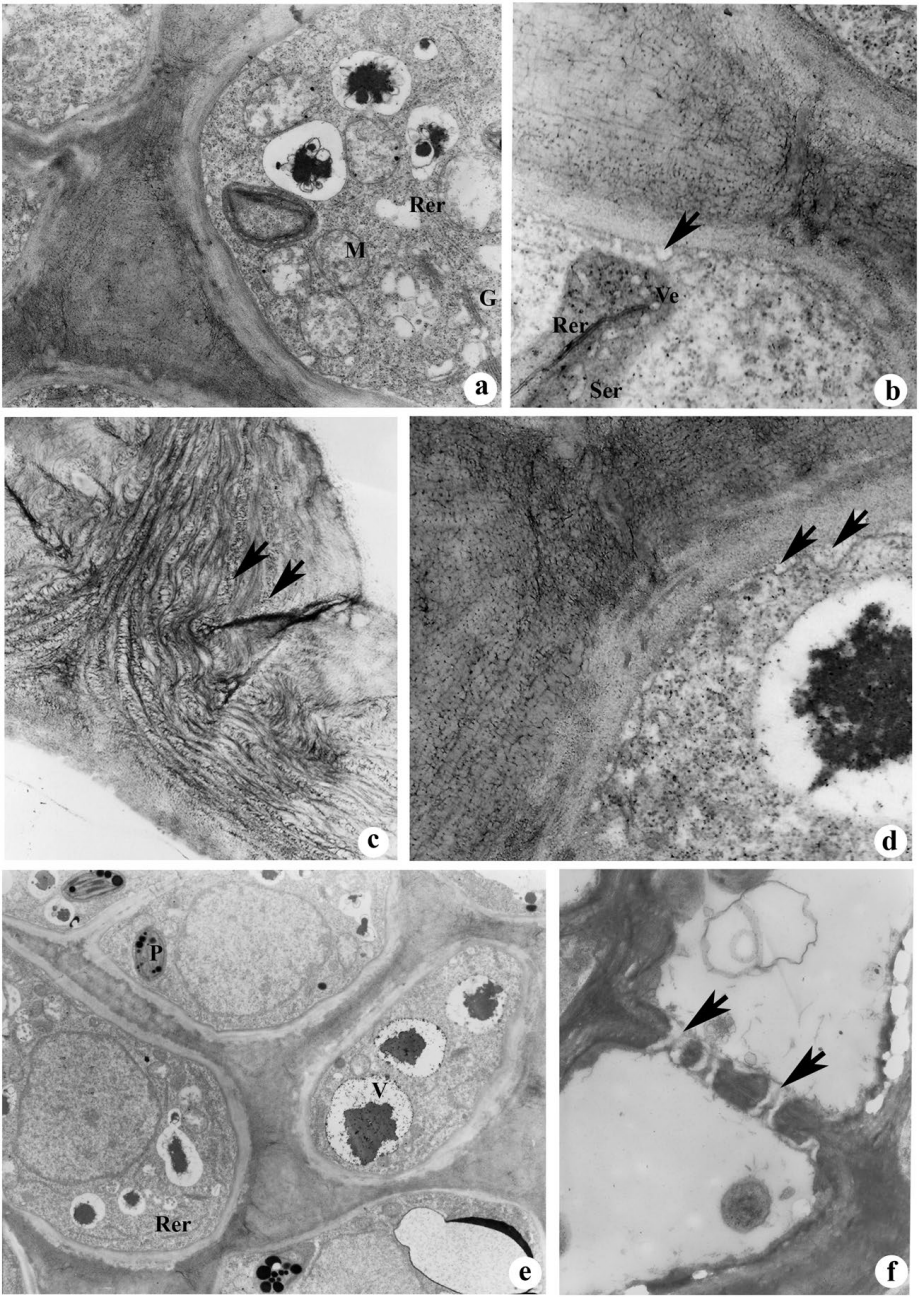
**a**–**f** TEM images of nectariferous cells showing ultrastructural changes during the secretory stage. **a** Nectariferous cells during the secretory stage showing the presence of numerous mitochondria (M) and rough endoplasmic reticulum (RER) near the cell wall. **b** Association of rough endoplasmic reticulum, smooth endoplasmic reticulum, and vesicles near the cell wall. Arrow indicates vesicles fused with the plasma membrane. **c** The loosely organized cell wall showing the presence of granular substances between microfibrils. **d** Presence of vesicular structures (arrows) in the space between the plasmalemma and cell wall. **e** Cells during the secretion stage showing a small-sized nucleus, rough endoplasmic reticulum (RER), and large vacuoles (V) containing osmiophilic substances. Note the absence of starch grains in plastids (P). **f** A sieve tube from phloem strand of nectary showing sieve plate with sieve pores and callose (arrows). Scale bar: **a**–**d** = 0.5 μm, **e**–**f** = 1 μm

The cell wall underwent drastic ultrastructural changes during this stage. Loosening of cellulose microfibrils followed by separation of middle lamellae resulted in the appearance of intercellular spaces and subcuticular spaces (Fig. 2e, f, g). The accumulation of nectar lead to further expansion of these spaces, giving the appearance of wide gap areas between cells (Fig. 2g). The plastids appeared darkly stained, fusiform to oval-shaped containing well-developed thylakoids and many plastoglobuli (Fig. 2h). Many of the vacuoles showed the presence of granular materials and osmophilic materials (Fig. 2h). The RER cisternae were unevenly dilated in the form of small rows and are often closely associated with plasmodesmata (Fig. 2h). The intercellular spaces showed the presence of granular and fibrillar secretions (Fig. 2h). An increase in the number of mitochondria with well-developed cristae was apparent during this stage (Fig. 3a). Association of rough ER, smooth ER (tubular), and secretory vesicles near the plasmalemma was noticed (Fig. 3b). Interestingly, the adjacent loosely organized cell wall area without plasmodesmata showed abundant accumulation of fibrillar secretions in the subcuticular spaces (Fig. 3b). The examination of loosely organized cell wall also revealed the presence of granular substances in the spaces between microfibrils (Fig. 3c). Vesicular structures were also noticed in space between plasmalemma and cell wall (Fig. 3d). Nucleus appeared smaller in size, while large vacuoles containing electron dense osmiophilic substances were noticed in the cytoplasm of subepidermal cells (Fig. 3e). Disappearance of starch from plastids was a conspicuous feature of nectariferous cells during the secretory stages (Fig. 3e). The sieve plate region of sieve cells connecting rachis to nectariferous cells showed the presence of callose in the sieve pore region (Fig. 3f).

### Late secretory stage

**Fig. 4:**
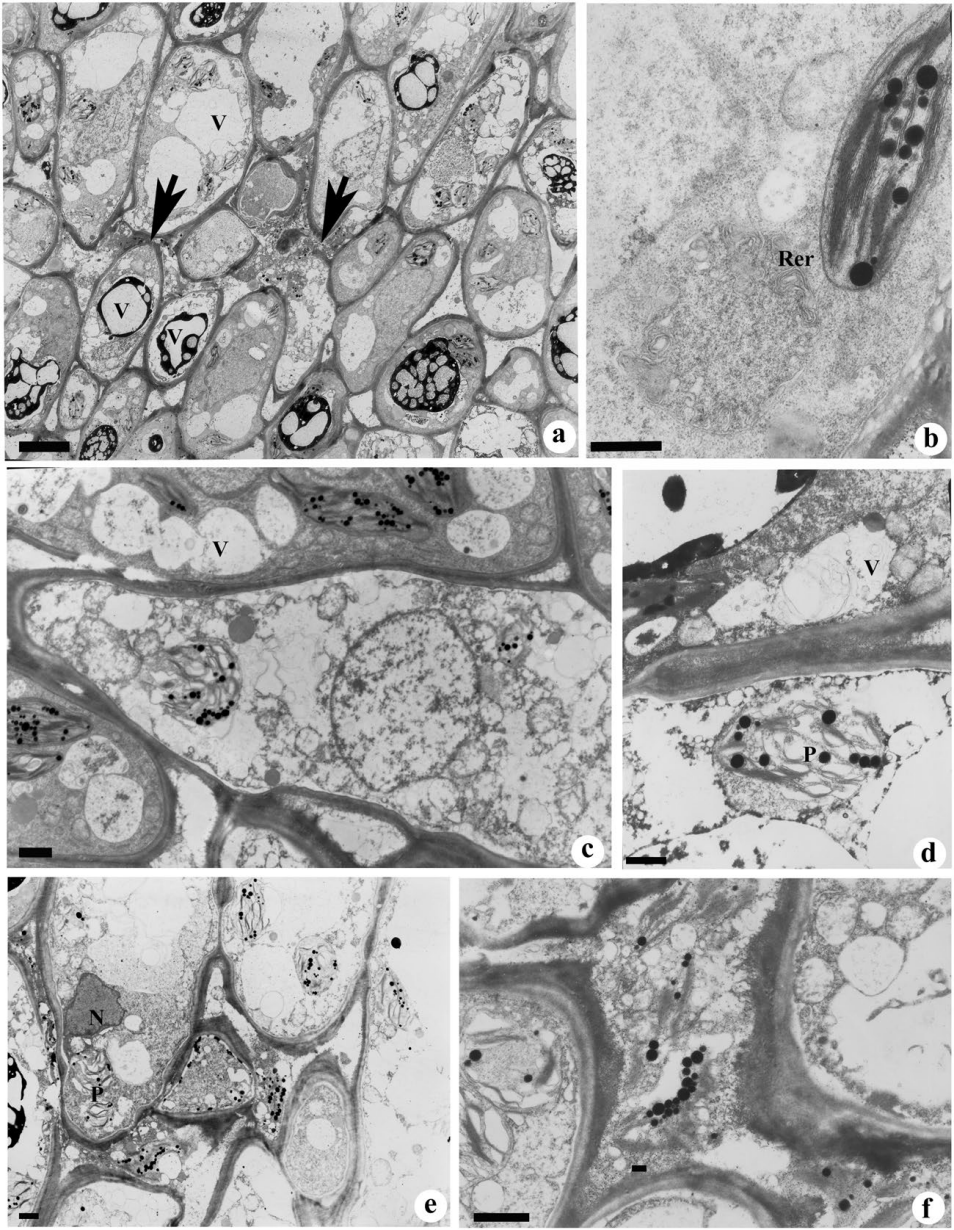
**a**–**f** TEM images of nectariferous cells showing ultrastructural changes during the late-secretory (**a**–**b**) and post-secretory stage (**c**–**f**). **a** Nectariferous cells showing degradation of the cell walls (arrows). Note the presence of large vacuoles. **b** A cell showing stalked rough endoplasmic reticulum (RER). Note the long and convoluted shape of ER cisternae. **c** Cells in the late secretory stage showing the presence of autophagous vacuoles (V) containing partially degraded cell organelles. **d** A disorganized plastid (P) showing a few membranes of thylakoids and plastoglobuli. **e**, **f** Disorganized nectariferous cells (**e**) with intercellular spaces (**f**) filled with cytoplasmic debris. Scale bar: **a** = 10 μm, **b**–**f** = 2 μm

This stage is characterized by the disintegration of nectariferous cells which occurs progressively towards the vascular bundle. Cell disintegration starts with the degradation of the cell wall (Fig. 4a). The adjacent cell below the degraded cell wall showed very dark cytoplasm (Fig. 4a). Cells often showed the presence of large vacuoles which occupies the majority of cell volume (Fig. 4a). Many of the vacuoles were free of osmiophilic substances, while few showed the presence of granular materials. Rough ER was stalked in layers, and ER cisternae were long and convoluted (Fig. 4b).

### Post-secretion stage

Extensive degradation of plastids, nuclei, and mitochondria was evident. Autophagous vacuoles containing partially degraded cell organelles were often noticed in the cytoplasm (Fig. 4d). Increase in vacuolar volume through the fusion of small vacuoles following more cell sap production was observed (Fig. 4c). The thylakoid structures of plastids were disorganized, while the presence of plastoglobuli was apparent even in the plastid lumen (Fig. 4d). The cytoplasmic contents released after cell wall degradation moved to intercellular and subcuticular spaces where it appeared as granular and floccular secretions (Fig. 4e, f).

## Discussion

The extrafloral nectary of *P. dulce* is an urn-like structure with a concave surface lined by epidermal cells. When the nectary is active, a drop of transparent and sticky nectar oozes out from the pit, and the dried nectar gives a brownish appearance to the mature nectary (Dave and Menon 1987). Similar type of cupular EFNs have been reported in *P. macradenium* and in closely related genus *Inga* which correlates with advanced nature of pollinia or polyads, and these specialized and unspecialized nature of foliar nectaries helps to determine the phylogenetic advancement of genera within mimosoid legumes (Elias, 1972). Elevated type of EFNs is found in 85 genera of legumes in which 81 belongs to Caesalpinioideae (Marazzi et al. 2019). The general histology of nectary in *P. dulce* is characterized by a single-layered epidermis with cuticle, nectariferous zone composed of outer parenchymatous and inner sclerenchymatous cells followed by highly vacuolated glandular cells. These anatomical characteristics in *P. macradenium* were described as indicators of foliar nectaries as highly specialized organs (Elias, 1972). The typical structural EFNs are characterized by the presence of parenchymatous nectariferous tissue composed of densely packed, thin-walled cells with dense cytoplasm and a glandular epidermal tissue through which exudation of nectar occurs (Marazzi et al. 2019). Single-layered non-secretory epidermal cells are characteristic features of EFNs from other reported species of the Leguminosae family (Pascal et al. 2000; Coutinho et al. 2012) and could be a taxonomic character for this family (Coutinho et al. 2022). Multilayered nectariferous parenchyma surrounded by a few layers of highly vacuolated cells where vascular tissues end is a common feature reported in the EFNs of Leguminosae (Durkee et al. 1999; Melo et al. 2010; Marazzi et al. 2013; Coutinho et al. 2022). The presence of two abaxial veins on either side of nectariferous cells making a continuous rim throughout the secondary rachis constitutes the vascular connection between EFNs and petiole in *P. dulce*. The vasculature in EFNs of *P. dulce* reported to be typical in its mode of branching pattern facilitating phloem connectivity for sugar supply to the secretary tissue at the tip of the nectary (Dave and Menon 1987). This type of vasculature pattern in *P. dulce* is an indicator of specialized EFN (Sharma and Pillai 1982; Dave and Menon 1987). A similar pattern of the vascular system comprised of the main vascular system of the petiole along with accessory bundles ensuring the exclusive supply of photoassimilates towards EFNs has been reported in *Chamaecrista* species (Coutinho et al. 2012, 2022).

In the majority of plants, the nectariferous cells (both floral and extrafloral) remain intact during the whole period of secretion (merocrine). The nectar secretion may occur through a transport pathway between nectariferous parenchyma to secretory cells or between nectariferous parenchyma to intercellular space and final exudation through modified stomata (Fahn 1987). On the contrary, the mature nectary in *P. dulce* showed nectar secretion associated with the dissolution of nectariferous cells. Hence, nectar secretion in *P. dulce* occurs in a holocrine manner. A similar kind of secretion has been previously reported in extrafloral nectaries of *Sambucus* sp. (Morini 1886), *Opuntia monacantha* (Daumann 1930), and *Sambucus nigra* (Fahn 1987).

The examination of changes in the cell wall and cytoplasm at different developmental stages of the nectariferous cells of *P. dulce* revealed characteristic features associated with the transport and secretion of nectar and subsequent autolysis of cells. During the pre-secretion stage, nectariferous cells showed dense cytoplasm rich in ribosomes, mitochondria, large nuclei often with more than one nucleoli, and plastids with accumulated starch grains. These cytological features indicate the active metabolic status of the cells. In poplar, the nectariferous parenchyma is distinguished from ground parenchyma by the presence of a dense granular cytoplasm rich in ribosome, mitochondria, and chloroplast suggesting high metabolic activity required for nectar production (Escalante-Perez et al. 2012). The presence of chloroplasts in the nectariferous cells is an indication of the contribution of plastids to the bulk of sugar secretion in floral nectaries (Pacini et al. 2003; Nepi 2007). In *P. dulce*, rough ER and smooth ER are abundantly found in the secretory stage. The RER is commonly attributed to the bulk production of protein (Werker and Voughan 1976), whereas SER has been attributed to lipid production in various secretory cells (Werker and Fahn 1981). In the present study, the occurrence of Golgi, secretory vesicles, ER, and plasmalemma near the plasmodesmata and the subsequent appearance of secretions in the intercellular and subcuticular spaces have been noticed during the secretory phase. Therefore, the abundance of ER cisternae which coincides with the onset of secretory activity suggests that ER is the principal organelle involved in secretion. The presence of vesicular structures between the cell wall and plasmalemma (multivesicular lomasomes) is likely to be related to the maintenance of constancy in the plasmalemma surface which plausibly increased during secretion (Bosabalidis and Tsekos 1982; Mohan and Inamdar 1986). These observations suggest that, in EFNs of *P. dulce*, granulocrine secretion is involved in the transfer of secretory materials to intercellular locations. The granulocrine secretion in poplar is marked by the presence of extrafloral nectary vesicles located in the outer apoplastic space as well as in the tip of the secretory cells (Escalante-Perez et al., 2012). The increase in the density of mitochondria and the disappearance of starch grains from plastids towards the secretion stage indicates increased carbohydrate metabolism and energy transfer in nectariferous cells (Findlay and Mercer 1971; Mohan and Inamdar 1986). The utilization of starch for the supply of sugar reserve to the nectary parenchyma makes the presence of chloroplasts even more meaningful in the supply of energy for nectar production (Coutinho et al. 2012).

One of the major ultrastructural changes observed in the nectariferous cell during the pre-secretory and secretory stages is the cell wall structure. The pre-secretory stage is characterized by cells with intact cell walls and small intercellular spaces. During advanced stages, the loosening of cellulose microfibrils and dissolution of middle lamellae leads to a wide gap between the cells (subcuticular space). These spaces are filled with granular and fibrillar secretions plausibly through the granulocrine mode of secretion. Similar events have been reported in extrafloral nectaries of *Plumeria* sp. (Mohan and Inamdar 1986) and are proposed to help in enhancing the effectiveness of secretion. The assembly of endomembrane components, RER, SER, secretory vesicles, and plasmalemma near the loosely organized cell wall area devoid of plasmodesmata and the presence of fibrillar secretions in the adjacent subcuticular space suggest the possible apoplastic secretory pathway independent of plasmodesmata. The granular secretory products may penetrate through the space between loosely organized cellulose microfibrils and enter into subcuticular space.

The late secretory stages of EFNs in *P. dulce* are characterized by an increase in vascular volume, a distinct increase in convoluted ER cisternae, autophagic vacuoles, and cell wall disintegration. The disintegration of the cell wall facilitates the wide passage for floccular secretion into the intercellular and subcuticular spaces. The pressure produced by the accumulation of secretory products in the subcuticular spaces in granulocrine mode and cell wall disintegration causes the cuticle to burst open, releasing the secretory material to the outside (Findlay and Mercer 1971). Figure 1c demonstrates that the mature nectaries in *P. dulce* shows breaks in the cuticle suggesting cuticular rupture might also facilitate the active secretion during late stages. The proliferation of the ER and autophagic vacuoles at the late stages of nectary development appears to be related to the lytic process of the disintegrating cells. An increase in ER cisternae at the late stages of secretion has been reported in septal nectaries of banana (Fahn and Banouaiche 1979) and in extrafloral nectaries of *Sambucus* (Fahn 1987). Cellular autolysis is believed to be initiated by the breakdown of the tonoplast. The appearance of autophagic vacuoles during the cessation of secretion indicates that they play a major role in the cellular breakdown and ensures post-secretory changes in the cell and finally the cell death (Butler and Simon 1971; Dodge 1971; Mohan and Inamdar 1986).

The results of the present study on the cellular mechanism of nectar secretion in advanced type of EFNs in *P. dulce* suggest that the symplastic continuity through ER, vesicles, and plasmalemma either with plasmodesmata or with loosely organized cell wall plays an important role in nectar secretion during early stages, while the advanced stage is characterized by typical holocrine mode where cell death, disintegration of cell wall, and rupture of cuticle facilitates the late stages of secretion.
